# Hyperthermic intravesical chemotherapy with mitomycin‐C for the treatment of high‐risk non‐muscle‐invasive bladder cancer patients

**DOI:** 10.1002/bco2.203

**Published:** 2022-12-02

**Authors:** Samantha Conroy, Karl Pang, Ibrahim Jubber, Syed A. Hussain, Derek J. Rosario, Marcus G. Cumberbatch, James W. F. Catto, Aidan P. Noon

**Affiliations:** ^1^ Academic Urology Unit, Department of Oncology and Metabolism University of Sheffield Sheffield UK; ^2^ Department of Urology Sheffield Teaching Hospitals NHS Foundation Trust Sheffield UK; ^3^ Academic Oncology Unit, Department of Oncology and Metabolism University of Sheffield Sheffield UK

**Keywords:** acceptability, high risk, hyperthermic intravesical chemotherapy, intravesical treatment, mitomycin C, non‐muscle‐invasive bladder cancer, tolerability

## Abstract

**Objectives:**

The objectives of the study are to explore tolerability, acceptability and oncological outcomes for patients with high‐risk non‐muscle‐invasive bladder cancer (NMIBC) treated with hyperthermic intravesical chemotherapy (HIVEC) and mitomycin‐C (MMC) at our institution.

**Patients and Methods:**

Our single‐institution, observational study consists of consecutive high‐risk NMIBC patients treated with HIVEC and MMC. Our HIVEC protocol included six weekly instillations (induction), followed by two further cycles of three instillations (maintenance) (6 + 3 + 3) if there was cystoscopic response. Patient demographics, instillation dates and adverse events (AEs) were collected prospectively in our dedicated HIVEC clinic. Retrospective case‐note review was performed to evaluate oncological outcomes. Primary outcomes were tolerability and acceptability of HIVEC protocol; secondary outcomes were 12‐month recurrence‐free, progression‐free and overall survival.

**Results:**

In total, 57 patients (median age 80.3 years) received HIVEC and MMC, with a median follow‐up of 18 months. Of these, 40 (70.2%) had recurrent tumours, and 29 (50.9%) had received prior Bacillus Calmette–Guérin (BCG). HIVEC induction was completed by 47 (82.5%) patients, but only 19 (33.3%) completed the full protocol. Disease recurrence (28.9%) and AEs (28.9%) were the most common reasons for incompletion of protocol; five (13.2%) patients stopped treatment due to logistical challenges. AEs occurred in 20 (35.1%) patients; the most frequently documented were rash (10.5%), urinary tract infection (8.8%) and bladder spasm (8.8%). Progression during treatment occurred in 11 (19.3%) patients, 4 (7.0%) of whom had muscle invasion and 5 (8.8%) subsequently required radical treatment. Patients who had received prior BCG were significantly more likely to progress (*p* = 0.04). 12‐month recurrence‐free, progression‐free and overall survival rates were 67.5%, 82.2%, and 94.7%, respectively.

**Conclusions:**

Our single‐institution experience suggests that HIVEC and MMC are tolerable and acceptable. Oncological outcomes in this predominantly elderly, pretreated cohort are promising; however, disease progression was higher in patients pretreated with BCG. Further randomised noninferiority trials comparing HIVEC versus BCG in high‐risk NMIBC are required.

## 1 INTRODUCTION

Bladder cancer (BC) is a common disease, accounting for 3% of all worldwide cancer diagnoses,^1^ of which three‐quarters of patients present with non‐muscle‐invasive disease (NMIBC).^2^ Due to variable recurrence rates,^3^ long‐term surveillance is required, and patients often require repeated surgical and intravesical treatments, making this one of the most expensive cancers to manage.^4^ Patients with high‐risk NMIBC (HR‐NMIBC), such as patients with high‐grade disease, lamina propria invasion, lymphovascular invasion, carcinoma‐in‐situ (CIS) or variant histopathology,^5^ need more aggressive management. First and foremost, accurate diagnosis by meticulous endoscopic resection, and re‐resection, is essential to ensure that there is no residual disease and that the tumour has been appropriately staged.^6^ Thereafter, patients may be offered either adjuvant intravesical therapy (usually Bacillus Calmette–Guérin [BCG]) or primary radical cystectomy (RC). The former is a bladder‐preserving strategy aiming to minimise recurrence and, more importantly, progression events,^7^, ^8^ while the latter is a more radical approach aiming to remove the bladder to irradicate those risks.^9^

Both intravesical BCG and primary RC have their limitations. Intravesical BCG can be poorly tolerated and fails to control local disease in up to a third of patients at 1 year,^10^ which can be associated with decision regret.^9^ Furthermore, BCG manufacturing limitations, exacerbated by the COVID‐19 pandemic, have led to BCG shortages, which have led to the rationing and reshaping of contemporary BCG schedules.^11^ Primary RC is a surgical procedure with associated morbidity, mortality and quality of life implications that must be carefully discussed with each patient. In patients whom BCG fails, who are unfit or unwilling to undergo RC, there is a paucity of effective alternative treatment options available to them in the clinic. Hence, there has been a drive to develop novel or alternative therapies to BCG treatment, which both are acceptable to patients and provide noninferior oncological outcomes.

One emerging treatment is intravesical hyperthermia combined with oncological agents—most commonly mitomycin‐C (MMC). Hyperthermia has been shown to synergistically interact with MMC to accelerate cancer cell death in vitro^12^; and in practical terms, enhances chemotherapy pharmacokinetics by improving MMC solubility and urothelial penetration.^13^

As a response to international BCG shortages, and lack of treatments available to patients in our region after BCG had failed, we introduced a hyperthermic intravesical chemotherapy (HIVEC) programme, combined with MMC, using the combat bladder recirculation system (BRS) device, into our specialist BC clinic. Here we present our single‐institution experience of using this treatment in an unselected, consecutive cohort of patients with HR‐NMIBC; the purpose of which was to evaluate the tolerability, acceptability and oncological efficacy HIVEC and MMC in patients with HR‐NMIBC treated at our institution.

## 2 METHODS

### 2.1 Patients

In the Department of Urology, Sheffield Teaching Hospitals NHS Foundation Trust, patients were considered for HIVEC and MMC treatment if they had failed ambient intravesical chemotherapy in whom BCG was contraindicated, for BCG unresponsive disease, or as a novel alternative to BCG considering ongoing concerns of international supply shortages. Patient demographics, instillation dates, and tolerability data were collected prospectively in our dedicated HIVEC clinic. Retrospective case note review was performed to evaluate oncological outcomes. Consecutive patients treated for HR‐NMIBC with HIVEC and MMC between August 2017 (when the clinic was established) and October 2019 were included.

At our institution, HR‐NMIBC is defined as patients with the following:

- New or recurrent tumours that are high‐grade (ISUP 1997/WHO 2004) or G3 (WHO 1973); any pT1 tumours; any evidence of CIS; or tumours with variant histopathological features.

In patients who had received prior BCG treatment, BCG failures were defined as per the 2017 EAU guideline on NMIBC (the guideline available at the time of project inception).^14^ EORTC risk scores were calculated for each individual patient, as per Sylvester et al. criteria.^15^

### 2.2 Intravesical MMC

Each intravesical MMC instillation was delivered using the Combat BRS device. Patients were catheterised with a 16F three‐way catheter, before delivery of 40‐mg MMC in 50 ml, which was heated externally to a temperature of 43 ± 0.5°C, using the combat BRS aluminium heat exchanger. Instillation time was 60 min. In the absence of evidence, risk‐stratified HIVEC instillation protocols were created, similar to that of the initial schedule of intravesical BCG; the rationale being that if there was a degree of immune‐modulation from HIVEC instillations, then a BCG‐adapted protocol would be appropriate. The HR‐NMIBC HIVEC protocol (6 + 3 + 3) consisted of six weekly induction instillations, followed by check cystoscopy; patients clear of disease were then offered two further maintenance cycles of three instillations (given weekly), with interval cystoscopy. Patients with disease recurrence during treatment, who were unwilling or unable to undergo RC were offered re‐induction (six further instillations).

### 2.3 Primary and secondary outcomes

The primary outcomes of the study were to evaluate the tolerability of HIVEC and MMC and acceptability of the proposed high‐risk protocol. Adverse events were recorded as per the common terminology criteria for adverse events (CTCAE) descriptors. Secondary outcomes were 12‐month recurrence‐free survival (RFS), progression‐free survival (PFS) and overall survival (OS).

### 2.4 Statistical analysis

Continuous variables are described using median with interquartile range (IQR) values, and categorical variables are described using counts and percentages. Kaplan–Meier method RFS, PFS and OS survival curves were generated using Prism v9.3.1. Exploratory subgroup analyses were performed using *χ* ^2^ and Fisher's exact tests, but were limited by sample size. Univariate analysis was performed using binary logistic regression for categorical binary outcomes to identify potential associated prognostic factors (including age, gender, previous recurrence or intravesical treatment, stage and EORTC scores) for disease recurrence, progression and survival. Statistical significance was defined as a *p* value <0.05.

## 3 RESULTS

### 3.1 Baseline patient demographics

A total of 57 patients were treated with HIVEC and MMC at our institution for HR‐NMIBC. Patient baseline demographics are shown in Table 1. The median (IQR) age of patients was 80.3 (76.5–86.0) years. As expected, many of the patients that referred for HIVEC and MMC were male (80.7%). Of the patients referred for HIVEC and MMC, 40 (70.2%) had recurrent tumours of which 32/40 (80.0%) previously harboured high‐grade disease, 12/40 (30.0%) were ≥pT1 tumours, and 8/40 (20.0%) had prior evidence of CIS. A review of the patients with recurrent tumours revealed that 32/40 (80.0%) had received at least one prior intravesical therapy and that 29/40 (72.5%) had received previous intravesical BCG (Table 1).

**TABLE 1:** HR‐NMIBC HIVEC cohort patient demographics and previous treatments received

		*n*	%
Age	Median (IQR)	80.3	(76.5–86.0)
	>70 years	50	87.7
Gender	Male	46	80.7
	Female	11	19.3
Previous treatment(s) if recurrent tumours	BCG only	24	60.0
	BCG + a‐MMC	3	7.5
	BCG + a‐MMC + epirubicin	1	2.5
	BCG + radiotherapy	1	2.5
	a‐MMC	2	5.0
	MMC + epirubicin	1	2.5
	No treatment	8	20.0
BCG status *n* = 29	Relapsing	8	27.6
	Refractory	10	34.5
	Unresponsive	5	17.2
	Intolerant	4	13.8
	None of the above	2	6.9

Abbreviations: a‐MMC, ambient mitomycin‐C; BCG, Bacillus Calmette–Guérin; HIVEC, hyperthermic intravesical chemotherapy; HR‐NMIBC, high‐risk non‐muscle‐invasive bladder cancer; IQR, interquartile range; MMC, mitomycin‐C.

### 3.2 Tumour characteristics at time of referral for HIVEC therapy

Table 2 reflects the histopathological characteristics at time of referral for HIVEC and MMC. Overall, 56 (98.2%) had high grade tumours; only one patient had a low‐grade tumour, which was classified as high‐risk NMIBC due to the presence of variant pathology. Ten (17.5%) patients had pT1 tumours, and 19 (33.3%) had evidence of CIS. Median (IQR) EORTC recurrence and progression scores were 5 (4–7.5) and 8,^5^, ^6^, ^7^, ^8^, ^9^, ^10^, ^11^ respectively.

**TABLE 2:** Index tumour characteristics at time of referral for HIVEC and MMC therapy

		*n*	%
Recurrent tumour	No	17	29.8
	Yes	40	70.2
Grade	LG^a^	1	1.8
	HG	56	98.2
Stage	pTis	6	10.5
	pTa	30	52.6
	pTa + CIS	11	19.3
	pT1	8	14.0
	pT1 + CIS	2	3.5
Total with CIS	Yes	19	33.3
	No	38	66.7
No of tumours	Single	29	50.9
	Multifocal	22	38.6
	CIS only	6	10.5
Size	<3 cm	46	80.7
	≥3 cm	11	19.3
EORTC recurrence score	0	0	0
	1–4	21	36.8
	5–9	32	56.1
	10–17	4	7.0
	Median (IQR)	5 (4–7.5)	
EORTC progression score	0	2	3.5
	2–6	20	35.1
	7–13	24	42.1
	14–23	11	19.3
	Median (IQR)	8 (5–11)	

Abbreviations: IQR, interquartile range; LG, low grade; HG, high grade; CIS, carcinoma in situ; EORTC, European Organisation for Research and Treatment of Cancer.

a LGpTa tumour with squamous variant pathology.

### 3.3 HIVEC protocol adherence and tolerability

Overall, 47 (82.5%) patients completed HIVEC induction. A total of 36 (63.2%) completed one cycle of HIVEC maintenance, and 19 (33.3%) completed the full 6 + 3 + 3 protocol. Reasons for not completing the protocol are depicted in Table 3. Disease recurrence (28.9%) and adverse events (28.9%) were the most common reasons for incompletion of protocol; interestingly, five (13.2%) patients opted to stop treatment due to logistical challenges with repeated hospital visits and time commitment of treatment. Adverse events were experienced in 20 (35.1%) patients throughout the treatment duration. The most common adverse events were allergic rash (10.5%), urinary tract infection (8.8%) and bladder spasm (8.8%). Adverse events led to incompletion of protocol in 11 (19.3%) patients, but were tolerable in the remainder.

**TABLE 3:** HIVEC protocol adherence and treatment tolerability

No. induction instillations	Median (IQR)	6 (6–6)	
Completion of induction	Yes	47	82.5
	No	10	17.5
Reinduction	Yes	6	10.5
	No	51	89.5
Number maintenance doses	Median (IQR)	3 (0–6)	
No. complete maintenance cycles	0	21	36.8
	1	36	63.2
	2	19	33.3
	3	3	5.3
Reason treatment not complete (*n* = 38)	Intolerability	11	28.9
	Recurrence	11	28.9
	Patient choice^a^	5	13.2
	Medical comorbidities/frailty	5	13.2
	COVID	2	5.3
	Death	2	5.3
	Other	2	5.3
Adverse events	No	37	64.9
	Yes	20	35.1
Adverse event description (*n* = 20)	Bladder spasm	5	8.8
	Dysuria	1	1.8
	Urinary frequency	2	3.5
	Urinary urgency	0	0.0
	Urinary incontinence	2	3.5
	Urinary tract pain	1	1.8
	Urinary tract infection	5	8.8
	Haematuria	1	1.8
	Rash	6	10.5
	Other	2	3.5

Abbreviation: IQR, interquartile range.

a For example, transport challenges to and from the department, finding the treatment too time consuming, other life commitments, opting for a watch and wait approach.

### 3.4 Clinical outcomes of HR‐NMIBC patients treated with HIVEC and MMC

**FIGURE 1:**
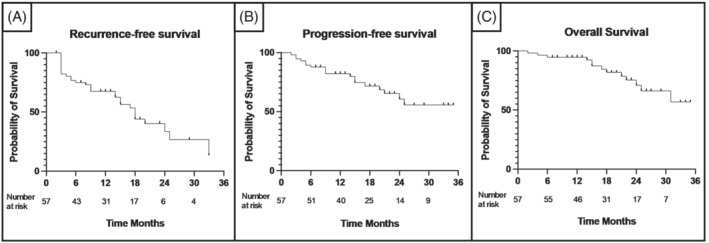
Kaplan–Meier survival curves depicting recurrence‐free survival (A), progression‐free survival (B), and overall survival (C) for HR‐NMIBC patients treated with HIVEC at our institution

At a median (IQR) of 18 (12.5–25) months, 25 (43.9%) patients experienced local recurrence, where 11 (19.3%) showed evidence of progression (three [5.3%] tumour grade progression and eight [14.0%] tumour stage progression). Median (IQR) time to recurrence and progression was 5 (3–14.5) and 9 months,^5^, ^6^, ^7^, ^8^, ^9^, ^10^, ^11^, ^12^, ^13^, ^14^, ^15^, ^16^, ^17^ respectively. Four (7.0%) patients progressed to muscle invasion (pT2 disease). Five (8.8%) patients required radical treatment, of which two had RC for recurrent high‐risk disease and three underwent external beam radiotherapy (EBRT) for muscle‐invasive (pT2) disease. One patient with progression to muscle‐invasive BC was treated with best supportive care. The 12‐month RFS, PFS and OS rates in this cohort were 67.5% (CI: 53.5–78.2), 82.2% (CI: 69.5–90.0), and 94.7% (confidence interval [CI] [84.6, 98.3]), respectively (Figure 1). Using multivariate analysis, no factors were identified as significant predictors of recurrence, progression or death in this cohort.

### 3.5 Subgroup analyses

We were unable to demonstrate significant difference in rates of recurrence or progression between patients with new and recurrent tumours (*p* = 0.15 and *p* = 0.15, respectively). Patients who had received prior BCG before HIVEC were not at higher risk of developing disease recurrence during treatment (*p* = 0.49) but were significantly more likely to develop progressive disease within the follow‐up period (31.3% vs. 7.1%, odds ratio [OR] 5.85, CI [1.2, 28.5], *p* = 0.04).

There were no differences in recurrence or progression risk between patients with and without CIS at time of referral (*p* = 0.85 and *p* = 0.74, respectively) nor was a difference demonstrated in terms of recurrence or progression between patients who completed the 6 + 3 versus 6 + 3 + 3 protocol (having removed patients who did not complete treatment protocol due to recurrence) (*p* > 0.99 and *p* > 0.95, respectively).

## 4 DISCUSSION

Here we present our single‐institution, real‐world experience of one of the larger described high‐risk‐only NMIBC cohorts treated with HIVEC and MMC.^16^, ^17^, ^18^, ^19^, ^20^, ^21^ Overall, HIVEC treatment served as a well‐tolerated and safe adjuvant treatment, with similar protocol completion rates to BCG^7^; hence, providing scope as a potential alternative candidate for HR‐NMIBC patients in an era of international BCG shortages, or indeed, as a salvage treatment in patients in whom BCG has failed. Oncological outcomes (RFS, PFS and OS) were promising in this high‐risk, pretreated cohort, and were similar to other high‐risk NMIBC HIVEC studies.^17^, ^18^

HR‐NMIBC represents almost one‐third of the BC workload managed at our institution,^22^ many of whom are unfit, or unwilling, to receive RC. Their management options are limited to adjuvant intravesical BCG,^8^ which has high failure rates due to toxicity and recurrence^7^, ^23^, ^24^; this is further compounded by poor BCG availability due to international supply shortages. Patients who develop recurrence during BCG treatment or who cannot tolerate the side‐effects, have sparse alternative effective options available in the clinic. One emerging treatment is intravesical hyperthermia with MMC. Hyperthemic MMC can be delivered using two models: radiofrequency‐induced thermo‐chemotherapy (RITE) using microwave radiation through a catheter device, and recombinant systems that use recirculation systems to externally heat and instil chemotherapeutic agents, such as, HIVEC.^25^

With recent evidence emerging to support the use of hyperthermic MMC, in both its forms, as a potential adjuvant treatment in high‐risk patients,^16^, ^17^, ^18^, ^19^, ^20^, ^21^, ^26^, ^27^ we established a specialist HIVEC clinic in our department. The primary focus was to explore the tolerability and acceptability of HIVEC treatment for patients with HR‐NMIBC. Our data align with the literature for HR‐NMIBC patients treated with HIVEC, where reported adverse events during treatment are common 33%–80%,^16^, ^17^, ^18^, ^19^, ^20^, ^21^ but are usually low grade (Grade 1–2), and often short‐lived (for example, bypassing and urinary frequency). In this cohort, 20 (35.1%) patients reported an adverse event (AE) during treatment, the most common of which were: rash, bladder spasm and urinary tract infection. AEs led to treatment cessation in 11 patients (28.9%), which is slightly higher than other reported studies (4%–28%)^16^, ^17^, ^18^, ^19^, ^20^, ^21^; this may suggest that older patient populations are less able to tolerate HIVEC instillations.

In terms of acceptability, the definitions of HIVEC incompletion are often inconsistently described in the literature and some report retrospective AE data collection, which could result in underreporting. In our study, we defined incompletion of HIVEC as the failure to complete the 6 + 3 + 3 high‐risk protocol. HIVEC induction was well tolerated (82.5% completion); however, only one‐third of patients completed the full protocol (comparable with that of BCG treatment^7^, ^24^). The most common reasons for lack of completion were disease recurrence and intolerability. It is worth noting that five (8.7%) patients reported that the travel burden to hospital, and time commitment required for repeated intravesical treatments, did not outweigh the benefits, and hence opted to discontinue treatment; this travel and time toxicity has also been described in BCG studies as a reason why patients stop treatment early,^24^ highlighting the importance of thorough patient counselling about logistics of treatment and the need for regular risk‐benefits review throughout the treatment course.

The secondary objective of this study was to explore oncological outcomes. As expected, recurrence events in this high‐risk, heavily pretreated cohort were common, affecting 43.9% of patients across a median follow‐up of 18 months. This likely reflects the natural history of the disease in a cohort with predominantly recurrent tumours (56.1%) and median EORTC risk score of five (1‐year recurrence risk of 35%–41%).^15^ Recurrences often occurred early in the disease course (median time to recurrence of 5 months), highlighting the need for rigorous high‐risk follow‐up protocols that include cystoscopic review. Recurrence (and progression) rates were not significantly higher in patients who received one versus two cycles of maintenance HIVEC; however, further prospective data are required to explore oncological outcomes using maintenance regimes of differential length.

In this study, disease progression was identified in 11 (19.3%) patients (median time to progression of 9 months), which was higher than expected. A median EORTC progression score of eight would usually confer a progression risk of 4%–7% at 12 months.^15^ The high progression rate in this study potentially reflects high levels of pretreatment in this group, which may alter the biology of the disease and subsequent treatment responses. It was most pronounced in patients pretreated with BCG who had a significantly higher risk of progression (*p* = 0.041); alternatively, it could reflect the nature of this cohort—with a median age of 80.3 years, it is likely that this group have higher competing morbidities, and hence, higher morbidity and mortality risk of curative primary RC.^28^, ^29^

Disease progression was identified and treated early (median time from progression to definitive treatment of 3 months), which was expedited by low thresholds for repeat tissue sampling, and early triage to radical therapy in those who were eligible. Only two patients who progressed despite BCG and HIVEC failure underwent RC, which likely reflects patients who initially opted for bladder‐preserving intravesical strategies, before proceeding with radical surgery as a last resort. Overall, the 12‐month RFS, PFS and OS rates were promising, with slightly higher rates than other high‐risk HIVEC studies.^17^, ^18^ Higher RFS, PFS and OS have been reported in one randomised trial of BCG versus HIVEC,^16^ which excluded patients with CIS, enrolled fewer patients with recurrent tumours or prior treatment, and had a lower median age; and the second, a large prospective observational cohort in Spain,^30^ which again had a lower median age difference (10 years younger), lower frequency of recurrent disease, and fewer pretreatment patients.

Therefore, in this single institution, real‐world study, we have demonstrated that HIVEC with MMC was safe, tolerable and had reasonable oncological outcomes in this elderly, un‐selected, high‐risk patient cohort. Limitations of this study include patient numbers, lack of prospectively collected clinical outcome data and that the experiences and outcomes from this study relate to a single, tertiary‐centre experience, which may not necessarily reflect broader national and international practices. Nonetheless, this study suggests that HIVEC with MMC may be a promising salvage therapy for patient whom BCG has failed, and who are ineligible or unwilling to undergo RC. Larger, Phase III prospective randomised trial data are required to build on the evidence from the HIVEC‐HR pilot Phase II trial,^16^ to definitively compare the tolerability and efficacy of HIVEC to BCG in HR‐NMIBC.

## 5 CONCLUSIONS

Our single‐institution experience suggests that intravesical HIVEC with MMC is deliverable, safe and tolerable. Oncological outcomes in this predominantly elderly, pretreated cohort are promising, but disease progression rates were higher in those pretreated with BCG. Further randomised trial data are required to definitively compare HIVEC to BCG for patients with HR‐NMIBC.

## CONFLICT OF INTEREST

There are no conflicts of interest to disclose.

## AUTHOR CONTRIBUTIONS

All authors prepared the manuscript and figures and approved the submitted manuscript.
